# Effects of Sex on Growth Performance, Carcass Traits, Blood Biochemical Parameters, and Meat Quality of XueShan Chickens

**DOI:** 10.3390/ani14111556

**Published:** 2024-05-24

**Authors:** Chunyou Yuan, Yong Jiang, Zhixiu Wang, Guohong Chen, Guobin Chang, Hao Bai

**Affiliations:** 1 Joint International Research Laboratory of Agriculture and Agri-Product Safety, Ministry of Education of China, Institutes of Agricultural Science and Technology Development, Yangzhou University, Yangzhou 225009, China; mz120201368@yzu.edu.cn (C.Y.); ghchen@yzu.edu.cn (G.C.); gbchang1975@yzu.edu.cn (G.C.); 2 College of Animal Science and Technology, Yangzhou University, Yangzhou 225009, China; jiangyong12126@163.com (Y.J.); wangzx@yzu.edu.cn (Z.W.)

**Keywords:** yellow-feathered chicken, sex, production performance, meat quality

## Abstract

**Simple Summary:**

Various kinds of yellow-feathered chickens are crossbred from Chinese local native chicken breeds and introduced broilers. The individual parts of yellow-feathered chickens are less suitable for sale after slaughtering than those of broilers, and they have mainly been sold as live whole chickens. With the establishment of cold chain logistics for chicken in China, chilled-type and processed yellow-feathered chicken products have appeared in the market. Xueshan chicken, a yellow-feathered chicken breed, which has high meat quality and good taste, is bred to meet the diversified needs of the market by Jiangsu Lihua Breeding Company. The present study used Xueshan chicken as an experimental model to determine the effect of sex on the slaughtering performance and meat quality. This study provides valuable breeding references for poultry producers by determining the basic production phenotypes of yellow-feathered chickens.

**Abstract:**

The demand for high-quality chilled chicken has continued to increase in China. Chickens are sexually dimorphic, and to better understand the specific differences in chicken production based on sex, we examined how sex affects growth performance, carcass traits, and meat quality of yellow-feathered chickens. Male and female Xueshan chickens were used as the experimental model. Although males exhibited better growth performance, including body weight (BW), body slope, keel, shank length, and shank girth (*p* < 0.05), as well as carcass traits, such as dressed weight, leg muscle, and lean meat, females had higher carcass and breast muscle yields (*p* < 0.05). Males had higher follicle density and yellowness (b*) of the skin and better skin than females (*p* < 0.05). Among blood biochemical parameters, the serum content of corticosterone (CORT) was higher in males, while those of superoxide dismutase (SOD), glutathione peroxidase (GSH-PX), total antioxidant capacity (T-AOC), and catalase (CAT) were lower in males than in females (*p* < 0.05). The pH levels, shear force, and moisture content quality were better in male breast meat, while the intramuscular fat content (IMF) was lower in males than in females (*p* < 0.05). The redness (a*) and moisture content were higher in male leg meat, while the pH, water-loss rate (WLR), lightness (L*), and IMF were lower (*p* < 0.05). The muscle fiber diameter and cross-sectional area were also higher in males (*p* < 0.05). Consumers felt that soup of male chicken was better than female (*p* < 0.05), while mouthfeel and tenderness acceptance of breast meat were different between the sexes. These results indicate that female chickens can be marketed as a whole carcass, while males are more suitable for processed carcass products. This study provides significant insights into the production and processing methodologies of yellow-feathered chickens.

## 1. Introduction

Chicken meat is an excellent and accessible source of protein, and high-yield chickens have become a source of low-cost protein, with the USDA (2024) reporting that chicken meat has been the most consumed meat worldwide for many years [1]. However, consumers are no longer satisfied with basic protein resources, and high-quality, healthy, diversified, and good-flavored chicken products are becoming more popular [2,3,4]. In contrast to European and American markets, which mainly distribute white-feathered broilers, both yellow-feathered chicken and white-feathered broilers are supplied by Chinese markets [5]. Processed chicken meat has become an inevitable trend in animal husbandry in China and has improved chicken production efficiency and provided high-quality products to consumers. In China, consumers prefer Xueshan chicken, a yellow-feathered chicken breed known for its high meat quality and good taste. It is widely reared in East China, with an annual output of more than 5-billion birds. Although this variety is mainly sold in the form of live birds, the production performance for slaughter and processing is currently being optimized for better sale. In recent years, owing to the negative impact of avian influenza and Newcastle disease, poultry production has gradually been converted from live chicken sale to scale breeding, centralized slaughtering, cold chain distribution, and chilled sale [5].

Chicken performance is influenced by breed [6], sex [7], nutrition [8], and feeding-management system [9]. Under the same genetic background and breeding methods, sex is the main factor influencing slaughter performance and meat quality [10]. In China, chicken soup from female yellow-feathered chickens represents a traditional and popular tonic, and consumers in different geographic regions tend to prefer local products [11]. Furthermore, consumers in different regions have different preferences for male and female chickens; a survey conducted by Jiangsu Lihua Company indicated that consumers in Northern and Southwestern China preferred large male chickens, whereas consumers in Southern and Eastern China preferred female chickens.

Antioxidant and anti-stress indicators in blood are also important parameters that usually reflect chicken performance and meat quality. Higher levels of antioxidant markers in the blood, such as total antioxidant capacity (T-AOC), catalase (CAT), and glutathione (GSH), are effective in enhancing meat color and tenderness [12]. Pre-slaughter treatments and conditions also significantly influence corticosterone levels and meat quality. For instance, pre-slaughter electroshock stunning has been shown to reduce corticosterone levels, thereby improving meat quality. Lower corticosterone levels are associated with better meat tenderness and reduced pH, enhancing overall meat quality [13].

Therefore, this study aimed to evaluate the effects of sex on the growth performance, carcass traits, and meat quality of yellow-feathered chickens. The findings may provide a reference for producers to rationalize their marketing strategies and maximize the economic value of their products according to sex characteristics and market demand.

## 2. Materials and Methods

### 2.1. Ethics Statement

All experimental procedures were approved by the China Council on Animal Care and the Ministry of Science and Technology of the People’s Republic of China. All experimental procedures were conducted in strict accordance with the Institutional Animal Committee of Yangzhou University (YZUDWSY).

### 2.2. Animals and Experimental Design

An improved strain of Xueshan chicken (slow-growing and slaughter-type breed) was bred by the Jiangsu Lihua Breeding Company, Changzhou, China. This breed is sold either after slaughter and division or as live whole chickens, which are usually marketed around 100 days of age [14,15]. Nine-hundred 1-day-old Xueshan chickens (half male and half female) were obtained from the Jiangsu Lihua Breeding Company, China. All chickens were reared in six identical pens (15 birds per square meter) for the first 27 days. At 27 days of age, chickens that died or had leg problems were excluded, and 180 chickens (with equal number of males and females) were randomly screened to meet the requirements and reared until 110 days. Groups of each sex consisted of three replicates (pens), with 30 chickens per pen. In addition, another 60 healthy chickens (half male and half female) were selected to be used to supplement the chickens culled during the breeding process and for the consumer acceptance test trials. The remaining chickens were subjected to commercial breeding and were no longer involved in the trial. All chickens that entered the test process had access to food and water (eight automatic drinking nipples and six feeders per pen. Chickens had free access to drinking clean and fresh water at all times and were fed with a standard commercial starter diet (1–28 days of age) (210.00 g/kg crude protein, 12.13 MJ metabolizable energy/kg) followed by a growing diet 1 (29–63 days of age) (185.00 g/kg crude protein, 12.55 MJ metabolizable energy/kg) and diet 2 (64–110 days of age) (160.00 g/kg crude protein, 12.97 MJ metabolizable energy/kg) (Table 1). The light scheme was 24 h on the first day and then reduced by 1 h every two days until 8 h of lighting were maintained each day (6 lux, 2700 K). The temperature was 24 °C, and the humidity was 55%. A 12 h fasting treatment was performed before each trial.

### 2.3. Growth Performance

The growth performance was evaluated based on several metrics: body weight, slope length from the shoulder to the ipsilateral ischial tuberosity, keel length of the front and rear ends of the keel, chest width as the distance between the two shoulder joints, chest depth from the first thoracic vertebra to the front keel, shank length from the supra metatarsal joint midway between the third and fourth toes, and shank grip as the circumference of the middle of the shin. At 28 and 110 days of age, all male and female Xueshan chickens were measured using a soft ruler (Deli, Ningbo, China) and weighed using a portable electronic scale (AISI BERG, Suzhou, China). All operations were performed in accordance with the standards issued by the Chinese Agricultural Standard 2020 [16].

### 2.4. Carcass Traits

Carcass traits include slaughter performance and carcass appearance. A total of 180 110-day-old male and female chickens were electrically stunned in a water tank (240 mA, 120 V, 5 s), slaughtered manually via a neck incision, and then depilated using an electric poultry depilator (TRANSAID, Shanghai, China). The de-feathered chickens were sent to a slaughtering room after preliminary treatments. For slaughter performance, carcass yield was measured as a percentage of live weight and semi-eviscerated weight (SEW), which is the weight of the carcass after the removal of the trachea, esophagus, crop, intestines, pancreas, spleen, gall bladder, reproductive organs, muscular gastric contents, and cuticular membranes. The eviscerated weight (EW) was the weight of the carcass after further removal of the heart, liver, glandular stomachs, lungs, and abdominal fat from the SEW. Breast muscle weight (BMW), leg muscle weight (LMW), and lean meat yield were expressed as percentages of the EW. For carcass appearance, follicle density, skin color, and the proportion of spotted skin were measured. Follicle density of the back midline and abdominal skin was calculated using a plastic template with hole measuring (2 × 2 cm^2^). Skin color, in terms of lightness (L*), redness (a*), and yellowness (b*), was measured using a colorimeter (CR-400; Konica Minolta, Tokyo, Japan) based on the standard formulated by the International Commission on Illumination. The colorimeter was calibrated before the experiment (L* = 99.41, a* = −0.07, and b* = −0.13). All operations were repeated three times, and the results were averaged (three replicates, with 30 birds per replicate). The percentage of spotted skin was recorded as S-, A-, B-, and C-grade chickens based on the skin condition on the back and sides of the chicken. Chickens that were similar to white-feathered broilers and had good carcasses without breakage or discoloration were rated as grade S (four points), chickens with a small amount of carcass discoloration were rated as graded A (three points), carcasses with a small amount of discoloration or broken skin were rated as graded B (two points), and those with large colors difference or skin breakage are rated as Grade C (one point). Ninety male chickens and 90 female chickens were scored and analyzed by non-parametric tests (Mann–Whitney U).

### 2.5. Blood Antioxidant and Anti-Stress Indicators

A negative-pressure blood-collection tube was used to collect approximately 5 mL of blood from the brachial vein of the chicken wing. The serum was separated using a refrigerated centrifuge at 4000 rpm for 5 min at 4 °C and stored at −20 °C for biochemical parameter determination of antioxidant and anti-stress indicators. The total antioxidant capacity (T-AOC), superoxide dismutase (SOD), glutathione peroxidase (GSH-PX), catalase (CAT), malondialdehyde (MDA), and corticosterone (CORT) contents were determined by enzyme immunoassay (ELISA) using an automatic enzyme immunoassay analyzer (Diatek DR200BS, Wuxi, China) and commercial ELISA kits (Beijing Sino-UK Institute of Biological Technology, Beijing, China). The creatine kinase (CK) content was determined using a colorimetric method with an automatic biochemical analyzer (Hitachi 7080, Tokyo, Japan) and a commercial kit (Biosino Bio-Technology and Science Incorporation, Beijing, China) (three replicates, with 30 birds per replicate).

### 2.6. Meat Quality

Breast and leg muscles were collected to determine meat quality via physicochemical properties (meat on the left) and nutritional composition (meat on the right). For physicochemical properties, pH values were obtained using a pH meter (pH-STAR; Matthaus, Berlin, Germany) after slaughter and labeled pH_1_ (muscle pH in 1 h) and pH_24_ (muscle pH after storage at 4 °C for 24 h). A small slit was cut into the meat, and the pH electrode tip was placed vertically to measure the pH three times, with the average of these three measurements determined. The shear force was calculated using a digital tenderness meter (C-LM3B; Tenovo Food, Beijing, China). The external fat and connective tissues were removed from the samples (length = 6 cm, width = 3 cm, and height = 3 cm), and the samples were separately packed in sealed bags and heated until the core temperature reached 70 °C. After cooling to room temperature (24 °C), shear force was measured three times using the digital tenderness meter to obtain the average value. The water-loss rate (WLR) was calculated using a meat-quality-pressure meter (Meat-1; Tenovo Food, Beijing, China), and samples were cut into small pieces (approximately 0.125 m^3^) in accordance with the standards issued by the China Agricultural Standard [17]. Skin color, including lightness (L*), redness (a*), and yellowness (b*), was measured using a CR-400 chroma meter. The shear force, WLR, and skin-color measurements were repeated three times and averaged. The proximate composition parameters, including the moisture, protein, intramuscular fat (IMF), and collagen, were measured using a near-infrared spectrophotometer after the samples (200 g) were stripped of exterior fat and connective tissue (FOSS FoodScan 78800; Hilleroed, Denmark) and processed by a grinder (three replicates, with 30 birds per replicate) [18].

To determine the characteristics of muscle fibers, the samples were subjected to hematoxylin-eosin (H&E) staining according to a previous method [19]. Breast and leg muscles (length = 6 cm, width = 3 cm, and height = 3 cm) were collected in individual 15 mL tubes, which were then filled with 4% paraformaldehyde for 24 h at room temperature. Paraffin-embedded samples were cut to a thickness of 5 µm using a cryostat (CM 1860; Leica Biosystems, Wetzlar, Germany) and a microtome (Leica Biosystems, Wetzlar, Germany). After drying overnight at 40 °C, the sections were counterstained with H&E. The processed samples were scanned using a Nikon 90i microscope (Nikon, Tokyo, Japan) at a magnification of 10 × 40, and the diameter (μm), area (μm^2^), and myofiber density were calculated using Image-Pro Plus 6.0 [20]. For each sample, five different points on five images containing approximately 250 muscle fibers were estimated.

Sensory analyses included chicken meat and chicken broth, with three parameters (mouthfeel, tenderness, and flavor) for chicken meat and three parameters (aroma, oiliness, and flavor) for chicken broth. Samples were obtained from a total of 30 chickens (males and females) in groups of five males or females, with three replicates of each sex (n = 3). The chicken-to-water weight ratio was set at 1:1.5, and the meat and broth samples were heated for 45 min using six induction cookers (Midea, Foshan, China, 2000 w) and maintained between 54 °C and 60 °C. Five grams of each sample were randomly assigned to 50 volunteers (n = 50). Each volunteer tasted each set of samples three times and averaged the results, scored on a 9-point scale (9 = extremely like, 5 = neither hate nor like, and 1 = extremely dislike).

### 2.7. Statistical Analysis

Data were statistically analyzed using SPSS software (version 22.0; SPSS Inc., Chicago, IL, USA). Before analysis, the normality of the data was verified using probability-probability plots. Tests for homogeneity of variances were performed based on ANOVA. Spotted skin level and sensory analyses were analyzed by the non-parametric test (Mann–Whitney U), and other data were analyzed by one-way ANOVA, and the significance of differences was tested using Duncan’s multiple comparison test. The data were assumed to be statistically significant when *p* < 0.05.

## 3. Results and Discussion

### 3.1. Growth Performance

The effects of sex on growth performance are presented in Table 2. Although most measurements, including body weight, body slope length, keel length, shank length, and shank girth, were higher in male chickens, compared with females (*p* < 0.05), whereas the chest width and depth were similar between the sexes (*p* > 0.05). Except for chest width and depth (*p* < 0.05), all measurements were correlated with age and sex. This trend of higher body weight and daily weight gain in male yellow-feathered chickens was consistent with that observed by Cygan–Szczegielniak et al and Motsepe et al. [7,8,9] for the Ross 308 variety.

Generally, male chickens possess a higher final body weight and larger body size than females, and significant sexual dimorphism is observed in different breeds [21,22,23]. While the chest width and depth of male chickens in this study were slightly greater than those of female chickens, other performance parameters were not significantly different. The male chickens in our study had longer shank lengths and girths, consistent with the observations of Charuta et al. [24], indicating that sex had an effect on shank development with respect to area, perimeter, and length. Intrinsic differences in body measurements between sexes directly affect other parameters of slaughter performance and can represent an effective predictive model for chicken production [25,26,27]. The results of our study implied that male chickens have better growth performance, greater size, and greater weight. Thus, they may have size advantages in the chilled chicken market when sliced and sold, such as larger chicken legs and wings. Producers can adjust production ratios according to the size differences between male and female chickens and provide consumers with diversified and economical choices.

### 3.2. Carcass Traits

Slaughter performance and carcass appearance traits were shown in Table 3 and Table 4, respectively. Slaughter performance is considered an important factor for evaluating the economic performance of animals and was thus evaluated in this study based on the sex of yellow-feather chickens. The associated parameters included the carcass yield, semi-eviscerated yield, eviscerated yield, breast muscle yield, leg muscle yield, lean meat yield, and dressed weight. The slaughter-performance traits listed in Table 3 are related to sexual dimorphism because they show that sex significantly affected carcass yield, breast muscle yield, leg muscle yield, lean meat yield, and dressed weight (*p* < 0.05). However, it did not significantly affect the semi-eviscerated and eviscerated yields (*p* > 0.05). Males had higher leg muscle yield, lean meat yield, dressed weight, and lower carcass and breast muscle yields, compared with females (*p* < 0.05). Although females had slightly higher semi-eviscerated yields and lower eviscerated yields, the differences were not significant (*p* > 0.05). Consistent with our findings, Nualhnuplong et al. [28] found that female Betong chickens had higher breast muscle yields than males, indicating that yellow-feathered hens may be better for producing processed chicken breast meat but not for producing whole chicken products. Moreover, our results were also consistent with those of the study by Fernandes et al. [29], who reported that male chickens (Cobb 500) had higher leg muscle yield and dressed weight than females. These results suggest that the choice between whole chicken sales and post-separation sales for chickens is related to sex. However, Ikusika et al. [30] observed that three strains of female chickens have higher dressed weights: ROSS, Aboaca, and Anak. These contradictory results may be attributed to the slaughter age and breed of the chickens. According to these studies, the differences in slaughter performance between sexes varied between breeds, with Cobb 500 male chickens presenting both higher leg muscle yield and dressed weight than females, and female ROSS chickens presenting higher dressed weights than males. Overall, the slaughter performance of males was better despite the higher female dressed weight. These sexual dimorphism differences were further demonstrated by the results of the present studies, which did not observe obvious slaughter performance advantages nor disadvantages of either sex of yellow-feathered chickens.

Carcass appearance is an important visual trait in chilled chicken markets because of the skin color, poultry integrity, and pore density, which directly influence consumer choices. As shown in Table 3, follicle density, skin color, and spotted skin proportion were used to assess the carcass appearance of yellow-feathered chickens in this study. The follicle density on both the back and abdomen of male chickens was significantly greater than that of females, and the a* value was higher in male chickens than in female chickens (*p* < 0.05). Moreover, the spotted skin level scores of male chickens were significantly higher than those of female chickens (*p* < 0.05), indicating that the carcass performance of male chickens was better. Chen et al., Ji et al., and Darnell D.K. et al. [31,32,33] found that the extensive activation of the Wnt/β-linked protein-signaling pathway in the feather capsules of yellow-feathered chickens may be influenced by nutritional levels. In this study, all chickens were reared under the same conditions. Therefore, the skin follicle density between males and females could have been related to sexual dimorphism. Although Wu et al. [34] reported that chicken skin color affects Chinese consumers’ choice, the popularity of the visually redder skin of male chickens was not investigated. Therefore, further trials are needed to elucidate the effect of skin color. According to Yuan et al. [15], male chickens have better skin color than females, which is consistent with our findings. Therefore, based on the results of this and previous studies, the carcass appearance of male chickens seems to be better than that of females.

### 3.3. Blood Antioxidant and Anti-Stress Indicators

The effects of sex on the blood biochemical parameters are shown in Table 5. Sex significantly affected serum biochemical parameters, including SOD, GSH-PX, T-AOC, CAT, and CORT (*p* < 0.05). Male chickens had higher serum CORT than female chickens, whereas female chickens had higher SOD, GSH-PX, T-AOC, and CAT levels than males (*p* < 0.05). The serum biochemical parameters MDA and CK were similar between male and female chickens (*p* > 0.05). Several studies [35,36,37,38] have reported indicators of antioxidant capacity and stress resistance in chickens, with SOD, GSH-PX, T-AOC, MDA, and CAT representing antioxidant indicators and CORT representing a stress resistance indicator. T-AOC refers to the total antioxidant capacity of all antioxidants in a sample and is a clinical biochemical index that reflects the total ability of the body to scavenge reactive oxygen species/nitric oxide synthase (ROS/NOS). SOD acts as a first line of defense against the harmful effects of intracellular oxygen radicals by catalyzing the disproportionate endogenous cytotoxic superoxide anion radicals to H_2_O_2_, which is then detoxified to water and oxygen by the combined action of CAT and GSH-PX [39]. The end-product of lipid peroxidation by free radical action in organisms is MDA, which is a reliable indicator of lipid peroxidation and an important indicator of antioxidant capacity in poultry [40,41]. The results of our study showed that female Xueshan chickens have better antioxidant capacity than males. Furthermore, the results of our study showed that female Xueshan chickens have better antioxidant and anti-stress capacity than males, which may also be the reason for the difference in meat quality. CORT is a glucocorticoid secreted by the adrenal cortex, and it is mainly responsible for sodium retention, potassium excretion, blood volume, and osmotic pressure regulation. Moreover, CORT is regulated by the body’s renin-aldosterone and angiotensin systems. Both Brown et al. and Zaytsoff et al. [42,43] found that higher CORT levels regulate physiological stress in chickens and further affect body tissues. These results demonstrate that male yellow-feathered chickens have a weaker antioxidant capacity and are more prone to stress than females. Thus, male chickens require additional management to avoid undesirable consequences, such as from stress. The stronger antioxidant capacity of female chickens can reduce the susceptibility of carcasses to oxidation, and the stress-resistance capacity is beneficial to reducing stress-induced effects during the slaughtering process, which can improve the marketability of intact carcasses.

### 3.4. Meat Quality

**Figure 1:**
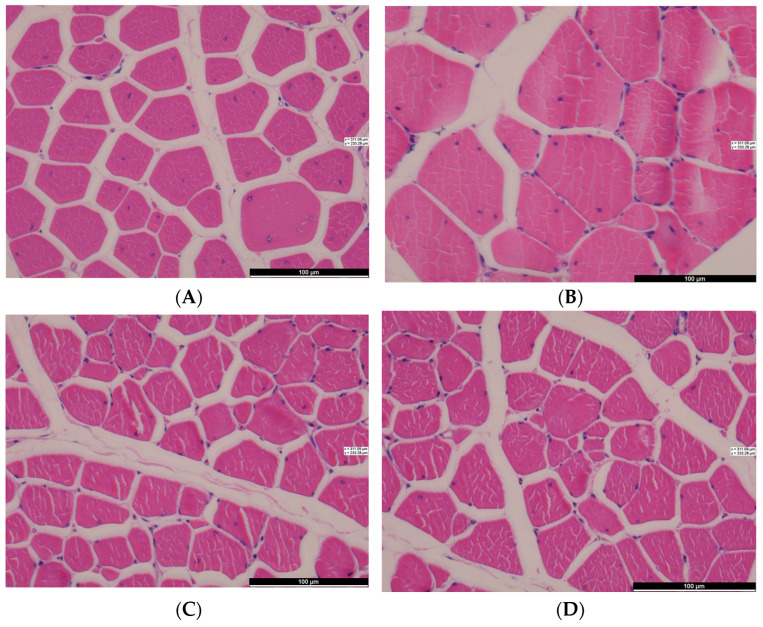
Microscopic picture of pectoral muscle of (**A**,**B**) male and (**C**,**D**) female chickens ((**A**,**C**) represent breast muscle, while (**B**,**D**) represent leg muscle.), hematoxylin and eosin (H&E) staining, magnification: 400*×*.

Meat quality is a decisive factor for chicken consumption. Currently, the tests for evaluating meat quality include muscle pH, WLR, meat color, shear force, nutrient content, fiber size, and consumer-sensory testing. The effects of sex on meat quality, muscle fiber, and sensory characteristics are shown in Table 6, Figure 1, and Table 7 and Table 8, respectively. pH is mainly influenced by phosphofructokinase activity, and the amount of lactic acid produced in the muscle is related to the amount of glycogen in the muscle [44]. Different treatments before slaughter can also affect the pH of the meat [45]. The pH_1_ and pH_24_ values of the male breast muscle were higher than those of the female breast muscle, and the pH levels of the leg muscle in both sexes were lower than those of the breast muscle (*p* < 0.05). Moreover, lower pH values were observed in male leg muscles relative to female leg muscles, which may be related to variations in muscle glycogen content during slaughter. All pH_24_ values were higher than the corresponding pH_1_ values, which was due to the accumulation of lactic acid in the muscles. Muscle tenderness is usually expressed as shear force, which is related to the diameter and cross-sectional area of the muscle fibers. Male chicken breast muscles had a greater shear force than female breast muscles (*p* < 0.05) because of the larger muscle diameter and cross-sectional area, as well as a lower density of male chickens. Huo et al. [46]. compared the difference in muscle fibers between slow-growing yellow chickens and fast-growing broilers and found that the faster the growth, the larger the diameter and cross-sectional area of the muscle fibers, which is consistent with the muscle traits of the fast-growing male chickens in this study.

WLR was used to evaluate the water-holding capacity of the muscle. The higher the WLR, the worse the water-holding capacity of the muscle, which means that nutrients and flavor substances are easily lost, thereby reducing the meat quality. The leg muscle WLR value of male chickens in this study was significantly lower than that of females (*p* < 0.05), indicating a greater water-holding capacity and better meat quality. An evaluation of the breast meat revealed that the sheer force of male chicken muscle was higher than that of females, suggesting that consumers may experience better tenderness with female chicken meat, which is consistent with the higher acceptance of female chickens (Table 8). IMF also has significant effects on the tenderness, juiciness, and flavor of chicken, which are important determinants of poultry meat quality [47,48]. Yellow-feathered chickens often have a higher IMF content than white-feathered broilers, which may explain the prevalence of consuming traditional native chickens in China because consumers think they are more nutritious and have better flavor than fast-growing chickens. In the sensory test, consumers felt that the breast meat and soup of male chicken were better than female, while tenderness acceptance of breast meat was significantly lower (*p* < 0.05). Among all chicken meat-quality evaluation metrics, color is one of the most significant factors that directly reflects the freshness of meat and induces purchasing desire [49]. The results of this study showed that the L* values of male chicken leg muscles were lower, and the a* values were higher compared to those of females. This indicates that male chicken leg meat is darker and redder, while female leg meat is brighter.

## 4. Conclusions

Our study evaluates the influence of sex on the growth performance, carcass characteristics, and meat quality of yellow-feathered chickens. Results indicate that male chickens demonstrate superior growth rates, slaughter performance, and carcass appearance, rendering them more appropriate for processed and segmented sales. In contrast, female chickens exhibit enhanced meat quality, making them ideal for whole carcass sales to achieve higher profits. Strategically aligning marketing approaches with these sex-specific traits and market demands can optimize economic returns. This research provides critical insights into the production and processing techniques for yellow-feathered chickens, offering a foundation for further optimization in the poultry industry.

## Figures and Tables

**Table 1:** Nutrient composition of experimental basal diets (%).

Items	1–28 d	29–63 d	64–110 d
Ingredient (%)			
Corn	55.82	62.49	69.30
Flour	2.00	2.00	2.00
Soybean meal	33.10	25.20	17.20
Corn protein flour	1.00	2.00	3.00
Soybean oil	1.31	1.96	2.27
Stone powder	1.41	1.24	1.25
Calcium hydrogen phosphate	1.36	1.11	0.98
Choline chloride	1.00	1.00	1.00
Premix	3.00	3.00	3.00
Nutritional level (%)			
Crude protein	21.00	18.50	16.00
Metabolizable energy (MJ/kg)	12.13	12.55	12.97
Ca	0.94	0.80	0.75
Available phosphorus	0.38	0.33	0.30
Digestible lysine	1.05	0.90	0.80

Premix provided for each kilogram of diet: V_A_: 7500 IU, V_D_: 3000 IU, V_E_: 50 IU, V_K3_: 50 mg; V_B1_: 90 mg; V_B2_: 300 mg; V_B6_: 60 mg; V_B12_: 0.4 mg; V_B3_: 1000 mg; V_B5_: 300 mg, Folate: 20 mg; Biotin: 2.0 mg; Fe: 1.3 g; Cu: 0.25 g; Zn: 2.0 g; Mn: 2.35 g; I: 20.0 mg; Se: 4.5 mg. Except for crude protein, whose value was measured, the levels of all nutrients were calculated 1.

**Table 2:** Effects of sex on growth performance of chickens at 110 days old.

	Sex			
Items	Male	Female	SEM	*p*-Value
Initial BW (g)	467.40 ^a^	418.40 ^b^	11.525	0.029
Initial Body slope length (cm)	12.43	12.18	0.108	0.257
Initial Keel length (cm)	6.12	5.96	0.060	0.193
Initial Chest width (cm)	4.88	4.74	0.036	0.063
Initial Chest depth (cm)	7.17 ^a^	6.85 ^b^	0.077	0.032
Initial Shank length (cm)	6.93 ^a^	6.62 ^b^	0.059	0.006
Initial Shank girth (cm)	3.15	3.01	0.039	0.075
Finial BW (g)	2526.0 ^a^	2204.9 ^b^	35.412	<0.001
Final Body slope length (cm)	25.31 ^a^	20.97 ^b^	0.215	<0.001
Final Keel length (cm)	18.70 ^a^	14.31 ^b^	0.249	<0.001
Final Chest width (cm)	8.58	8.21	0.138	0.198
Final Chest depth (cm)	9.48	9.22	0.115	0.267
Final Shank length (cm)	9.02 ^a^	7.69 ^b^	0.100	<0.001
Final Shank girth (cm)	5.05 ^a^	4.29 ^b^	0.049	<0.001
Daily BW gain (g)	25.10 ^a^	21.79 ^b^	0.432	0.001
Daily Body slope length gain (mm)	1.57 ^a^	1.07 ^b^	0.023	<0.001
Daily Keel length gain (mm)	1.53 ^a^	1.01 ^b^	0.028	<0.001
Daily Chest width (mm)	0.45	0.42	0.017	0.408
Daily Chest depth (mm)	0.28	0.29	0.019	0.847
Daily Shank length (mm)	0.26 ^a^	0.13 ^b^	0.012	<0.001
Daily Shank girth (mm)	0.23 ^a^	0.15 ^b^	0.007	<0.001

Significance: ^a,b^ *p* < 0.05; data represent three replicates, with 30 birds per replicate; SEM: standard error of the mean; final growth performance at 110 D; daily growth performance gain was calculated from 28 to 110 D.

**Table 3:** Effects of sex on slaughter performance of chickens at 110 days old.

	Sex			
Items	Male	Female	SEM	*p*-Value
Carcass yield (%)	85.61 ^b^	87.74 ^a^	0.499	0.044
Semi-eviscerated yield (%)	79.37	80.06	0.660	0.608
Eviscerated yield (%)	67.51	66.50	0.591	0.400
Breast muscle yield (%)	18.18 ^b^	21.01 ^a^	0.355	0.001
Leg muscle yield (%)	25.89 ^a^	21.41 ^b^	0.257	<0.001
Lean meat yield (%)	44.07 ^a^	42.43 ^b^	0.372	0.037
Dressed weight (g)	2197.58 ^a^	1859.46 ^b^	42.410	0.001

Significance: ^a,b^ *p* < 0.05; data represent three replicates, with 30 birds per replicate; SEM: Standard error of the mean.

**Table 4:** Effects of sex on carcass appearance of chickens at 110 days old.

		Sex			
Items		Male	Female	SEM	*p*-Value
Follicle density (piece/cm^2^)	Back	4.21 ^a^	3.56 ^b^	0.075	<0.001
	Abdomen	3.59 ^a^	2.86 ^b^	0.052	<0.001
Skin color	L*	69.14	67.61	0.323	0.021
	a*	7.90 ^a^	5.18 ^b^	0.220	<0.001
	b*	17.19	15.68	0.461	0.108
Spotted skin level	Points	3.33 ^a^	2.93 ^b^	0.887	*p* < 0.001 Z = −3.535

Significance: ^a,b^ *p* < 0.05; data represent three replicates, with 30 birds per replicate; SEM: Standard error of the mean. L*: Lightness; a*: Redness; b*: Yellowness.

**Table 5:** Effects of sex on blood biochemical parameters of chickens at 110 days old.

	Sex			
Items	Male	Female	SEM	*p*-Value
SOD (U/mL)	84.54 ^b^	102.49 ^a^	2.906	0.021
MDA (nmol/mL)	2.41	2.03	0.083	0.059
GSH-PX (U/mL)	357.61 ^b^	386.03 ^a^	5.569	0.043
T-AOC (U/mL)	12.30 ^b^	13.80 ^a^	0.298	0.045
CAT (U/mL)	65.13 ^b^	84.85 ^a^	1.076	<0.001
CORT (ng/mL)	4.84 ^a^	3.70 ^b^	0.178	0.019
CK (U/L)	1555.05	1531.03	134.923	0.923

Significance: ^a,b^ *p* < 0.05; data represent three replicates, with 30 birds per replicate; SEM: Standard error of the mean. Total antioxidant capacity (T-AOC); Superoxide dismutase (SOD); Glutathione peroxidase (GSH-PX); Catalase (CAT); Malondialdehyde (MDA); Corticosterone (CORT); Creatine kinase (CK).

**Table 6:** Effects of sex on meat quality of chickens on Day 110.

Items	Sex	pH		Shear Force (N)	Water Loss Rate (%)	Meat Color			Proximate Composition			
		pH_1_	pH_24_			L*	a*	b*	Moisture (%)	Protein (%)	Intramuscular Fat (%)	Collagen (%)
Breast muscle	Male	6.32 ^a^	6.31 ^a^	15.42 ^a^	10.13	47.44	12.72	12.81	72.19 ^a^	25.97	0.64 ^b^	0.25
	Female	6.17 ^b^	6.13 ^b^	12.49 ^b^	9.50	48.01	15.27	14.36	71.53 ^b^	26.02	1.00 ^a^	0.24
	SEM	0.019	0.013	0.512	0.714	0.956	0.764	0.863	0.097	0.107	0.057	0.030
	*p*-value	<0.001	0.002	0.008	0.664	0.769	0.107	0.378	0.002	0.789	0.004	0.931
Leg muscle	Male	5.96 ^b^	5.93 ^b^	19.59	13.14 ^b^	54.51 ^b^	3.06 ^a^	14.68	74.74 ^a^	21.87	2.89 ^b^	0.52
	Female	6.39 ^a^	6.17 ^a^	19.60	17.19 ^a^	58.26 ^a^	1.00 ^b^	16.59	73.10 ^b^	21.88	4.01 ^a^	0.47
	SEM	0.056	0.030	1.250	0.529	0.484	0.269	0.576	0.160	0.096	0.158	0.029
	*p*-value	0.001	<0.001	0.997	0.001	0.001	0.001	0.110	<0.001	0.934	0.001	0.437

Significance: ^a,b^ *p* < 0.05; data represent three replicates, with 30 birds per replicate; SEM: Standard error of the mean. L*: Lightness; a*: Redness; b*: Yellowness. pH_1_: pH value measured 1 h after slaughter. pH_24_: pH value measured 24 h after slaughter. Water loss rate, % = (W Initial − W Final)/W initial × 100.

**Table 7:** Effects of sex on muscle fiber characteristics of chickens on Day 110.

		Sex			
Items		Male	Female	SEM	*p*-Value
Breast muscle	Diameter (μm)	52.32 ^a^	46.66 ^b^	0.667	<0.001
	Cross-sectional area (μm^2^)	2318.03 ^a^	1656.17 ^b^	37.781	<0.001
	Density (piece/mm^2^)	319.21 ^b^	494.09 ^a^	27.554	0.013
Leg muscle	Diameter (μm)	56.59 ^a^	43.21 ^b^	0.987	<0.001
	Cross-sectional area (μm^2^)	2759.09 ^a^	1306.46 ^b^	51.140	<0.001
	Density(piece/mm^2^)	236.52 ^b^	398.09 ^a^	21.031	0.005

Significance: ^a,b^ *p* < 0.05; SEM: Standard error of the mean.

**Table 8:** Sensory characteristics of chicken’s breast and soup.

		Sex				
Items	Mean Rank	Male	Female	SEM	*Z*-Value	*p*-Value
Breast meat	Mouthfeel	7.82 ^a^	7.37 ^b^	0.90	−2.980	0.003
	Tenderness	7.16 ^b^	7.76 ^a^	0.99	−3.052	0.002
	Flavor	7.69	7.43	0.91	−1.432	0.152
Soup	Aroma	8.51	8.39	0.77	−1.276	0.202
	Oiliness	6.37	6.27	1.51	−0.557	0.578
	Flavor	8.33 ^a^	7.94 ^b^	0.50	−3.585	<0.001

Significance: ^a,b^ *p* < 0.05; (mean rank, n = 50). SEM: Standard error of the mean.

## Data Availability

All available data are incorporated in the manuscript.
